# Effect of Fluorinated Graphite (FG) Addition on Friction Performance of FG-Ni/WC/CeO_2_ Cladding Layers over a Wide Temperature Range

**DOI:** 10.3390/ma18173983

**Published:** 2025-08-25

**Authors:** Ouyang Li, Guirong Yang, Wenming Song, Ying Ma

**Affiliations:** 1 School of Materials Science and Engineering, Lanzhou University of Technology, Lanzhou 730050, China; 2 Machinery Industry Shanghai Lanya Petrochemical Equipment Inspection Ltd., Lanzhou 730070, China

**Keywords:** fluorinated graphite, wide temperature range, vacuum cladding, CeF_3_, wear mechanisms

## Abstract

This study fabricated fluorinated graphite (FG)-reinforced Ni/WC/CeO_2_ cladding layers on 45 steel substrates using vacuum cladding technology. Their microstructure, phase composition, mechanical properties, and tribological behavior over a wide temperature range (25–800 °C) were systematically characterized. The results demonstrate that FG addition promotes the formation of a self-lubricating CeF_3_ phase. The optimal CeF_3_ phase formation efficiency occurred at a 1.5 wt% FG content (NWF15). The NWF15 cladding layer exhibited the smallest average grain size (15.88 nm) and the lowest porosity (0.1410%) among all samples. Mechanical testing revealed that this cladding layer possessed the highest microhardness (1062.7 ± 21.9 HV_0.2_). Its H/E and H^3^/E^2^ ratios, indicative of resistance to elastic strain and plastic deformation, reached 0.0489 and 0.0291, respectively. Tribological tests revealed pronounced temperature-dependent wear behavior: abrasive wear was predominant at 25 °C; adhesive wear dominated from 200 to 600 °C; and oxidative wear became the primary mechanism at 800 °C. Throughout this temperature range, the CeF_3_ phase effectively reduced wear damage by suppressing groove propagation and providing effective lubrication, particularly under high-temperature conditions.

## 1. Introduction

Material degradation induced by high-temperature wear constitutes a major challenge for high-performance mechanical systems, such as aerospace propulsion systems [1] and heavy manufacturing equipment [2,3]. The economic and safety impacts stemming from component failure due to friction and wear are severe, driving continuous research into advanced protective cladding layers. Nickel-based alloys have attracted extensive attention and research due to their excellent high-temperature strength, oxidation resistance, and elevated-temperature wear resistance [4,5,6,7,8,9]. However, under prolonged high-temperature service conditions, nickel-based alloys are susceptible to high-temperature softening, which compromises their service life. To enhance the hardness of nickel-based alloys, researchers often employ strategies such as incorporating hard-phase particles and refining grains [10,11,12,13,14,15,16,17,18,19]. For instance, Wang [20] fabricated a Ni-30 wt% WC cladding layer via laser cladding technology. The hardness of this cladding layer was 2.5 times that of the 27SiMn steel substrate, while its wear rate was significantly reduced to 1.9% of the substrate’s wear rate. Similarly, Zhu [21] introduced cerium oxide (CeO_2_) into a Ni60A-35 wt% WC cladding layer to achieve grain refinement. The results demonstrated that compared to the CeO_2_-free Ni60A-35 wt% WC cladding layer, the addition of 0.5 wt% CeO_2_ increased the hardness by a factor of 1.62 and reduced the wear volume by 23.4% during friction testing at 600 °C. Although increasing hardness does enhance the wear resistance of nickel-based alloys, an excessive pursuit of high hardness often promotes the formation of pores and cracks within the cladding layer. This defect formation may counteract or even outweigh the wear resistance benefits gained from high hardness.

The incorporation of solid lubricants into nickel-based alloys can significantly enhance their wear resistance [22,23,24,25,26,27,28]. For instance, Shi [29] fabricated a Ni_3_Al alloy reinforced with WS_2_, Ag, and h-BN solid lubricants using spark plasma sintering (SPS) technology. The results indicated that Ag and WS_2_ served as effective lubricants at relatively low temperatures, whereas h-BN exhibited significant lubricating effects at elevated temperatures. Although Ag and h-BN can provide synergistic lubrication within the temperature range from room temperature to 800 °C, the presence of Ag reduces the overall hardness of the alloy, and the addition of h-BN increases its porosity. These side effects adversely affect the wear resistance of the alloy.

To address the limitation that externally added solid lubricants struggle to provide effective lubrication continuously across a wide temperature range, researchers have adopted strategies involving the in situ formation of solid lubricants [30,31,32] to effectively extend their operational temperature range. Carbon nanomaterials (e.g., graphite derivatives) commonly function as reinforcement phases and solid lubricants in composites. Fluorinated graphite (FG) [33] exhibits superior thermal stability to pristine graphite, owing to its distinctive C-F bonds, along with favorable lubricating properties. Notably, the fluorine in FG can react with cerium oxide (CeO_2_ [34,35,36,37]) under specific conditions, presenting a potential mechanism for in situ CeF_3_ generation. The lamellar hexagonal structure of CeF_3_ facilitates shear sliding, rendering it suitable as a high-temperature solid lubricant. Nevertheless, the controlled synthesis of CeF_3_ through FG incorporation into Ni/WC matrices, optimization of FG content to maximize CeF_3_ generation and microstructural refinement, and systematic evaluation of the resultant cladding layers’ tribological behavior across a wide temperature range (RT-800 °C) remain unexplored.

This study employed vacuum cladding technology to fabricate FG-Ni/WC/CeO_2_ cladding layers with varying fluorinated graphite (FG) mass fractions (0.5, 1.0, 1.5, and 2.0 wt%) on a 45 steel substrate. The phase composition, mechanical properties, and tribological behavior over a temperature range of 25–800 °C of the cladding layers were systematically investigated, elucidating the evolution of wear mechanisms with temperature and the friction-reducing mechanism contributed by the in situ-formed CeF_3_, thereby providing important theoretical and experimental insights for improving the wear resistance of nickel-based cladding layers over a wide temperature range.

## 2. Experimental Procedure

### 2.1. Material Preparation

**Figure 1:**
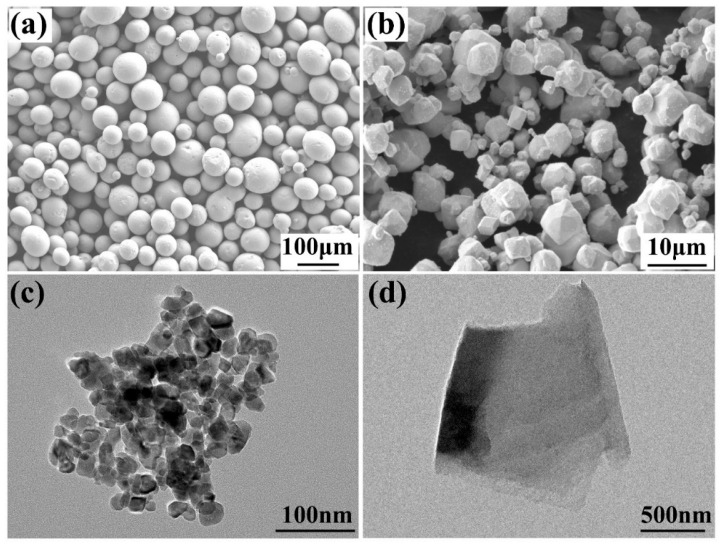
(**a**) SEM morphology of nickel-based alloy; (**b**) SEM morphology of WC; (**c**) TEM morphology of CeO_2_; (**d**) TEM morphology of FG.

The 45 steel substrate (50 mm × 40 mm × 10 mm) was sequentially polished with 60# to 2000# sandpapers to remove oxides, ultrasonically cleaned in anhydrous ethanol, and dried. The nickel-based alloy’s particles had a particle size of 150 mesh, with its chemical composition shown in Table 1, and the WC particles had a particle size of 1800 mesh. The morphology of the nickel-based alloy particles is shown in Figure 1a, and that of the WC particles in Figure 1b. Both materials were supplied by Buwei Applied Materials Technology Co., Ltd., Shanghai, China. TEM images of CeO_2_ (average particle size 50 nm) and fluorinated graphite (FG) (36.54 g/mol) are presented in Figure 1c,d, provided by Casting and Research Metal Material Co., Ltd., Hebei, China.

**Figure 2:**
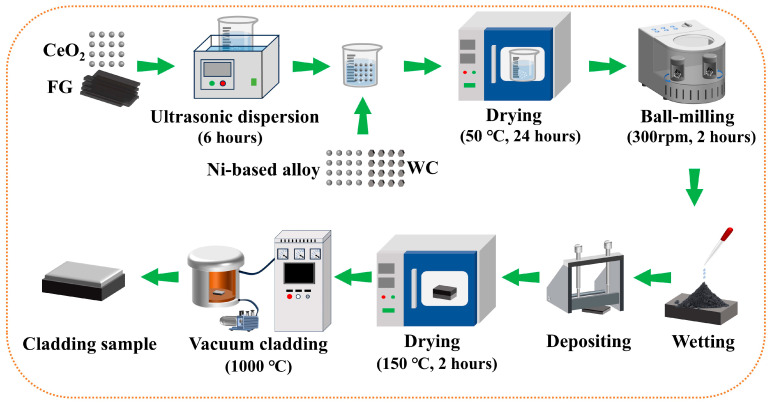
Schematic diagram of sample preparation.

Our prior work on CeO_2_-Ni/WC cladding layers demonstrated that the layer with 0.5 wt% CeO_2_ exhibited superior mechanical properties and room-temperature wear resistance. Based on these findings, the CeO_2_ content for the current FG-Ni/WC/CeO_2_ cladding layers was set at 0.5 wt%. To ensure the uniform distribution of FG and CeO_2_ within the cladding layer, pre-weighed FG and CeO_2_ were placed in absolute ethanol. The mixture was subjected to ultrasonic dispersion for 6 h at a frequency of 80 kHz using a Model KQ-100E ultrasonic cleaner (Kunshan Ultrasonic Instrument Co., Ltd., Kunshan, China) to promote homogeneous dispersion. After ultrasonic treatment, pre-weighed Ni-based alloy particles and WC particles were added dropwise to the resulting suspension and thoroughly mixed. Subsequently, the mixture was placed in an electric drying oven and dried at 50 °C for 24 h. Subsequent preparation steps followed the method described in Ref. [38]. The preparation flowchart is shown in Figure 2. Based on the weight fraction of the FG, the fabricated cladding layers were designated as NWF5, NWF10, NWF15, and NWF20, with their specific compositions shown in Table 2.

### 2.2. Material Characterization©

Using Electrical Discharge Wire Cutting (EDWC) technology, a metallographic sample with dimensions of 10 mm × 10 mm × 10 mm was cut from the cladding layer. Metallographic specimens were mechanically ground with 80# to 2000# SiC abrasive papers and polished with a 0.5 μm diamond suspension. Phase composition was characterized by a D/max-2400 X-ray diffractometer (Rigaku, Tokyo, Japan) with Cu Kα radiation (λ = 0.15406 nm), scanning from 10° to 90° at 2°/min. Thermogravimetric analysis (TGA) of nano-CeO_2_ and -FG was performed using a TGA 4000 instrument (PerkinElmer Inc., Shanghai, China). The measurements were performed under a N_2_ atmosphere with a heating rate of 10 °C/min, utilizing sample masses of approximately 10 mg. Porosity was quantified via threshold segmentation in Image-Pro Plus 6.0 (Media Cybernetics, Rockville, MD, USA), following ASTM E2109. A Quanta 450-FEG scanning electron microscope (SEM, FEI, Hillsboro, OR, USA) equipped with an energy-dispersive X-ray spectroscopy (EDS) system was used to characterize the morphology of the cladding layer and its worn surfaces.

### 2.3. Nanoindentation and Microhardness Test

Surface nanohardness and elastic modulus of the cladding layers were characterized via a KLA G200 nanoindenter (KLA Corporation, Milpitas, CA, USA) in continuous stiffness measurement (CSM) mode using a diamond tip (150 nm radius) with a 10 mN peak load at a 0.5 mN/s loading rate. Vickers microhardness was measured on surfaces and cross-sections via a W1102D37 tester (Sauer-Danfoss Co., Ames, IA, USA) compliant with ASTM E384 (200 gf load, 10 s dwell time, 100 μm intervals). All measurements were triplicated to ensure data accuracy.

### 2.4. Friction Test

Friction tests were performed using a ball-on-disk configuration under dry sliding conditions with an HT-1000 tribometer (Lanzhou Zhongke Kaihua Technology Development Co., Ltd., Lanzhou, China). A Si_3_N_4_ ball with a 6 mm diameter (hardness: 1800 HV; elastic modulus: 300 GPa) was employed as the counter body. Experimental parameters included a 10 N applied load, 561 rpm rotational speed, 4 mm wear track radius, and 30 min test duration; temperatures were set at 25 °C, 200 °C, 400° C, 600 °C, and 800 °C. When the temperature was stabilized at the target value and held for 10 min, the friction test commenced. To eliminate surface roughness effects, specimen surfaces were pre-polished to achieve Ra values of 0.05–0.08 μm. Wear volume loss and track morphology were analyzed using a 3D interferometric surface profilometer (Rtec UP-2000, San Jose, CA, USA), while post-test Si_3_N_4_ counter balls were examined via optical microscopy (OM). Phase composition on wear tracks was characterized using ATR3110 Raman spectroscopy (Optosky, Xiamen, China, laser wavelength: 532 nm; spot size: 1 μm).

## 3. Results and Discussion

### 3.1. Phase Composition and Micromorphology

**Figure 3:**
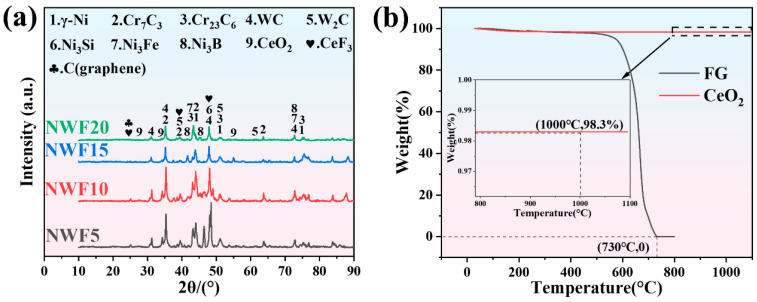
(**a**) XRD patterns of FG-Ni/WC/CeO_2_ cladding layers; (**b**) TGA of CeO_2_ and FG. The region marked by the dashed box corresponds to the magnified subplot in (**b**), highlighting local features of the TGA curve.

Figure 3a shows the XRD diffraction patterns of the cladding layers with different FG contents. Peaks below 5% intensity correspond to trace Ni-borides or instrumental artifacts, with a volumetric fraction <0.3% (per Rietveld refinement), exerting no significant impact on properties. The main phases present in the cladding layer, identified based on the ICDD PDF-4+ 2023 standard for phase analysis, include the following: γ-Ni (00-004-0850), Cr_7_C_3_ (00-036-1482), Cr_23_C_6_ (00-035-0783), WC (00-051-0939), W_2_C (00-035-0776), Ni_3_Si (00-065-1984), Ni_3_Fe (00-038-0419), and Ni_3_B (00-048-1419) [38]. In addition to the aforementioned phases, the XRD pattern revealed faint diffraction peaks at 2*θ* = 28.5°, 33.1°, and 47.5°, attributed to the (111), (200), and (220) planes of CeO_2_ (00-043-1001), respectively. Peaks corresponding to the (110), (300), and (220) planes of CeF_3_ (00-032-0199) were observed at 2*θ* = 24.5°, 39.1°, and 48.5°. CeF_3_ exhibits outstanding wide-temperature-range self-lubricating properties. Its hexagonal crystal structure facilitates easy slip along the basal (001) planes under shear stress. At elevated temperatures, fluoride ions exhibit enhanced activity and migrate, leading to the formation of a CeF_3_ protective film at the friction interface, effectively minimizing wear [39]. A thermogravimetric analysis (TGA) of CeO_2_ and FG is presented in Figure 3b. CeO_2_ exhibited a weight loss of ~1.7% at 1000 °C, whereas FG underwent complete decomposition at 730 °C. Based on the XRD and TGA results, the following reactions are inferred to occur during the vacuum cladding process:

2CeO_2_ → 2Ce_2_O_3_ + O_2_ (1)

2FG → 2G + F_2_ (2)

Ce_2_O_3_ + 3F_2_→2CeF_3_ + O_2_ (3)

**Figure 4:**
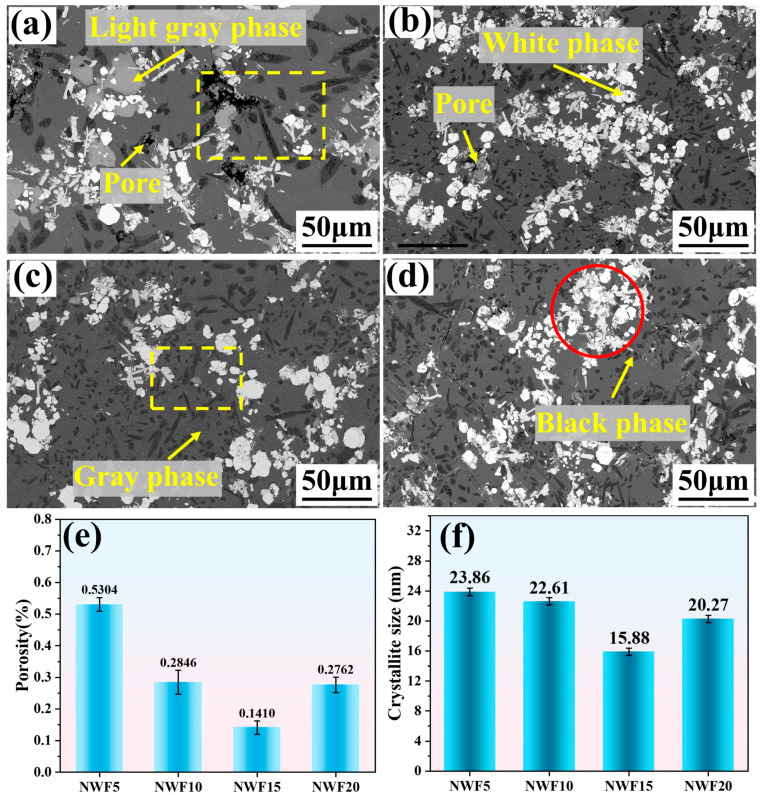
(**a**–**d**) Grain morphology of FG-Ni/WC/CeO_2_ cladding layers: (**e**) porosity; (**f**) crystallite size.

Figure 4a–d show the microstructures of the NWF5, NWF10, NWF15, and NWF20 cladding layers, which primarily consist of black needle-shaped, gray matrix, light gray blocky, and white particulate phases, along with sparse porosity. With increasing FG content, the maximum length of the black acicular phase in the clad layer gradually decreased, from (54 ± 6) μm (NWF5) and (46 ± 7) μm (NWF10) to (36 ± 6) μm (NWF15) and finally to (27 ± 3) μm (NWF20). Simultaneously, the area fraction of the black acicular phase (calculated using Image-Pro Plus 6.0 software) gradually increased, from 7.12% (NWF5) and 9.63% (NWF10) to 10.12% (NWF15) and finally to 13.52% (NWF20). Additionally, the white particulate phase underwent a transition from a dispersed state to an agglomerated state, as indicated by the region marked with a red circle in Figure 4d. Quantitative analysis (Figure 4e) indicates that porosity first decreases and then increases, with a minimum value of 0.1410% at NWF15 and a maximum value of 0.5304% at NWF5. Average grain sizes (Figure 4f) were calculated using the Scherrer equation [40] based on XRD peaks at 2*θ* = 31.2–72.8°. The formula *D* = 0.89*λ*/(*β* cos *θ*) (*λ*: X-ray wavelength; *β*: FWHM; *θ*: diffraction angle) yielded values of 23.86 nm for NWF5, 22.61 nm for NWF10, 15.88 nm for NWF15, and 20.27 nm for NWF20. The Ce:F molar ratios, calculated based on Reaction (3) stoichiometry (Ce:F = 1:6), were 1:2.35 for NWF5, 1:4.71 for NWF10, 1:7.06 for NWF15, and 1:9.42 for NWF20. NWF15 (Ce:F = 1:7.06) closely approximated the ideal ratio, where uniform FG dispersion enhanced heterogeneous nucleation, resulting in minimized grain size (15.88 nm) and porosity (0.1410%). In NWF5 (0.5 wt% FG), insufficient additives led to inhomogeneous distribution, weakening nucleation efficacy and increasing both grain size (23.86 nm) and porosity (0.5304%). For NWF20 (2.0 wt% FG), excessive FG induced melt splashing due to intensified F_2_ evolution (Reaction (2)), disrupting solidification and resulting in grain coarsening to 20.27 nm with increased porosity.

**Figure 5:**
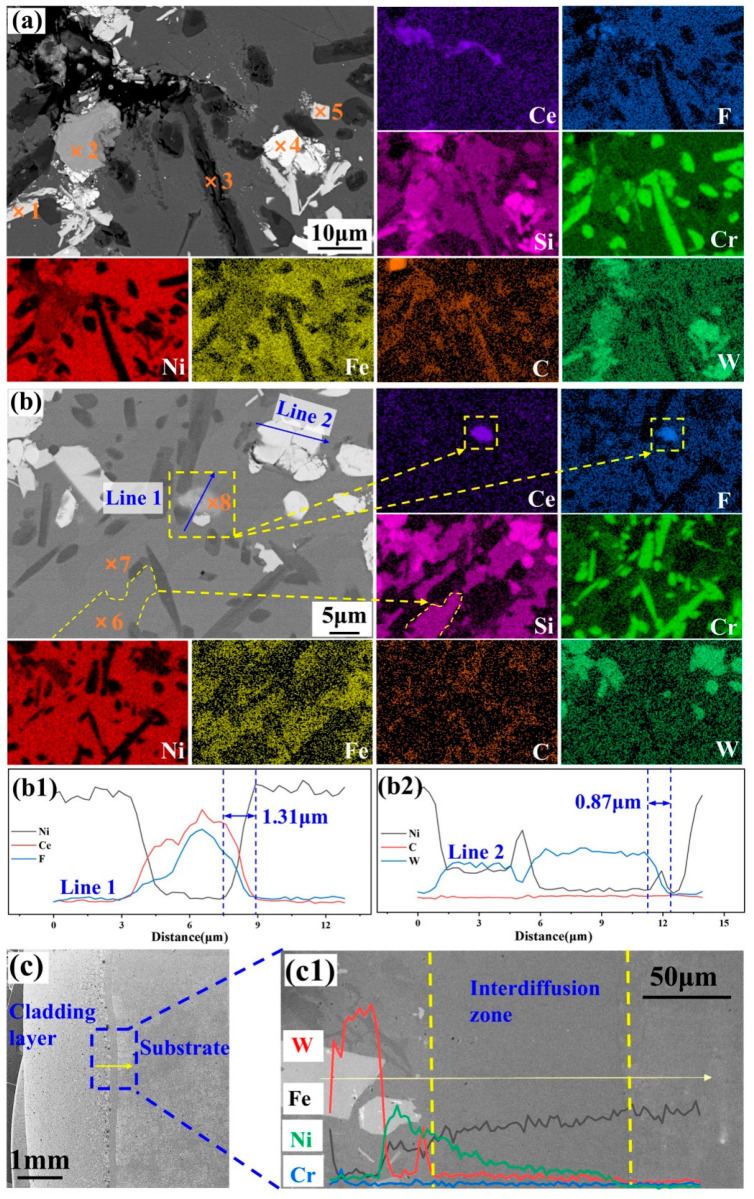
(**a**) SEM and EDS analysis of the rectangle area in Figure 4a; (**b**) SEM and EDS analysis of the rectangle area in Figure 4c; (**b1**) elemental line scan (Line 1) from Figure 4b; (**b2**) elemental line scan (Line 2) from (**b**); (**c**) cross-sectional morphology of NWF15; (**c1**) EDS elemental line scanning results of (**c**).

To further analyze the phase and elemental composition of the cladding layer, EDS scanning of the surface was performed. Figure 5a,b present magnified images of the rectangular areas in Figure 4a,b, respectively. Comparative analysis reveals that the black needle-like phase is primarily composed of Cr and C, the gray matrix phase is mainly composed of Ni and Fe, and the white granular phase consists mainly of W and Si. The EDS point scan data in Figure 5a,b are presented in Table 3. By analyzing the atomic percentage of the elements and combining the XRD and EDS surface scan results, the phase at point 1 is determined to be predominantly Ni_3_B and WC. The phases at points 2 and 5 are predominantly Ni_3_B. The black needle-like phase at point 3 is mainly composed of Cr_7_C_3_ and Cr_23_C_6_. The main phase at point 4 is WC. The phase at point 6 is primarily Ni_3_Si. Point 7 is mainly composed of γ-Ni, Ni_3_Fe, and Ni_3_B. Point 8 has higher concentrations of F and Ce elements, and the main phase is CeF_3_. With increasing FG content, the maximum length of the Cr_7_C_3_ and Cr_23_C_6_ phases gradually decreased, while their area fraction increased, suggesting that FG inhibited the growth of the Cr_7_C_3_ and Cr_23_C_6_ phases. Furthermore, the agglomeration of the WC and W_2_C phases indicates that the escaping gases (primarily F_2_, see Equation (2)) generated during the cladding process by excessive FG addition (2.0 wt%) adversely affected the homogeneous dispersion of the phases.

Figure 5(b1,b2) present the EDS line scan results of Line 1 and Line 2 in Figure 5b. The phase passed by Line 1 mainly consists of the in situ-generated CeF_3_. The diffusion depth of CeF_3_ in the nickel-based alloy is about 1.31 μm. The phase passed by Line 2 mainly consists of the added WC, and the diffusion depth of the WC particles in the nickel-based alloy is about 0.87 μm. This suggests that the in situ-generated CeF_3_ exhibits greater stability in the nickel-based alloy. Figure 5c presents the cross-section morphology of NWF15. From left to right, the sequence consists of the cladding layer, interdiffusion zone, and substrate, with an average cladding thickness of 1.92 mm. EDS line scanning analysis revealed that the elemental interdiffusion depth at the interface was approximately 110 μm, confirming the formation of a metallurgical bond between the substrate and the cladding layer. Compared to the 40 μm of the Ni/30%WC cladding layer [38], this diffusion depth represents a significant increase of 175%. CeO_2_ facilitates the diffusion pathways by reducing the interfacial energy and refining the grains [41], while fluorinated graphite decomposition generates F radicals that remove interfacial oxides and induce lattice distortion [42]. The synergistic interplay between these components results in a significant enhancement of the diffusion depth to 110 μm.

### 3.2. Nanoindentation and Microhardness Analysis

**Figure 6:**
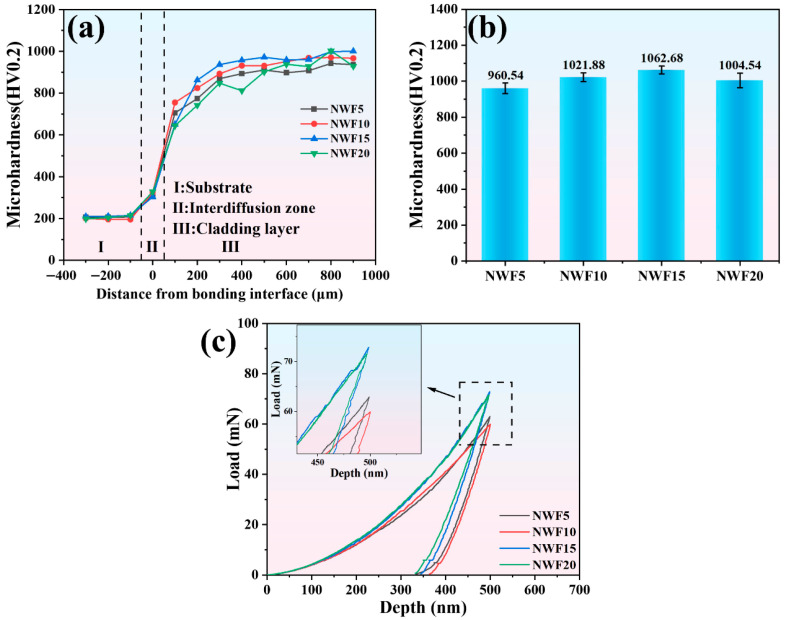
(**a**) Cross-sectional microhardness distribution of cladding layers; (**b**) surface microhardness of cladding layers; (**c**) load–indentation depth curves of cladding layers.

Figure 6a,b comparatively present cross-sectional/surface microhardness distributions of cladding layers with varied FG contents. The substrate (45 steel) exhibits a hardness of 205.6 ± 6.5 HV_0.2_, while the interdiffusion fusion zone reaches 312.4 ± 8.9 HV_0.2_. Cladding hardness initially increases then decreases with rising FG content: 960.5 ± 29.5 HV_0.2_ for NWF5, rising to 1021.9 ± 24.0 HV_0.2_ (NWF10), peaking at 1062.7 ± 21.9 HV_0.2_ (NWF15), and declining to 1004.5 ± 40.5 HV_0.2_ for NWF20. This hardness enhancement correlates significantly with reduced porosity and grain size, originating from synergistic strengthening effects of porosity diminution and grain refinement. Figure 6c presents the nanoindentation load–displacement curves for the clad layers with different FG contents. The average nanohardness (H), elastic modulus (E), H/E, and H^3^/E^2^ values are summarized in Table 4. The H/E ratio reflects the fracture toughness, with a higher value indicating that the cladding layer is less prone to crack formation during friction. The H^3^/E^2^ ratio characterizes the resistance to plastic deformation, with a higher value signifying enhanced resistance against penetration by the Si_3_N_4_ counter ball during friction [10,43]. Among the cladding layers with varying FG contents, NWF15 exhibits the highest H/E and H^3^/E^2^ ratios, of 0.0489 and 0.0291, respectively, consequently suggesting superior tribological performance. Mechanical properties are synergistically governed by microstructure and phases: The minimum grain size (15.88 nm, Figure 4f) and optimized CeF_3_ distribution (Figure 5(b1)) in NWF15 enable peak hardness (1062.7 HV_0.2_) and high H^3^/E^2^ (0.0291 GPa) via Hall–Petch strengthening and crack suppression. Reduced porosity (0.141%, Figure 4e) further minimizes stress concentration.

### 3.3. Friction and Wear Analysis

**Figure 7:**
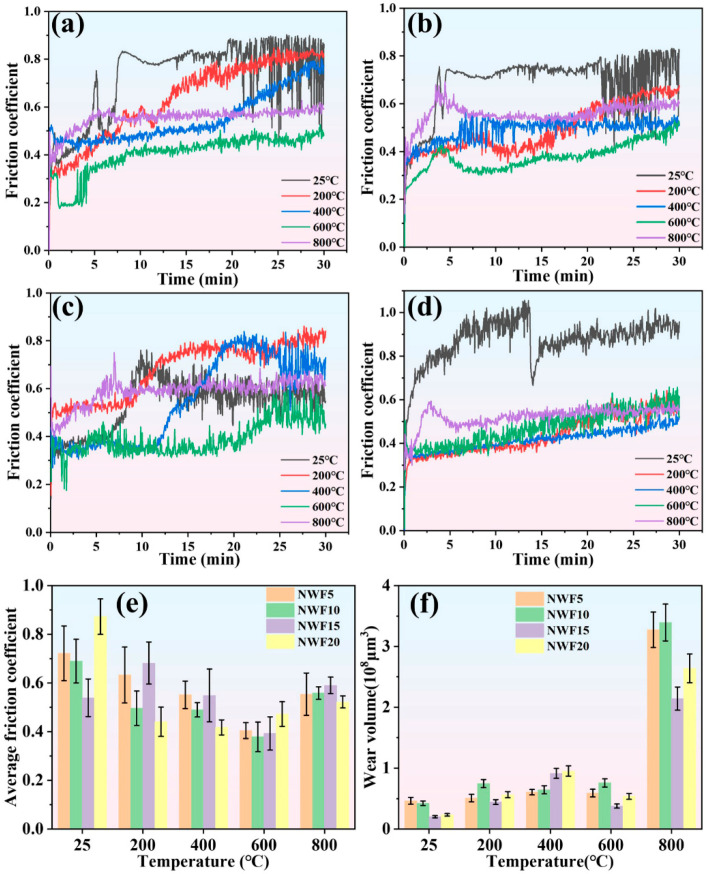
Friction coefficient curves of (**a**) NWF5, (**b**) NWF10, (**c**) NWF15, and (**d**) NWF20. (**e**) Average coefficient of friction; (**f**) wear volume.

Figure 7a–d depict the evolution of the friction coefficient for the NWF5, NWF10, NWF15, and NWF20 cladding layers within the temperature range of 25–800 °C. Within the 25–600 °C range, the friction coefficient curve exhibits three distinct stages: (1) initial running-in stage: the friction coefficient increases linearly; (2) mid-term steady stage: the friction coefficient remains stable with minor fluctuations; (3) later destabilization stage: the friction coefficient increases significantly and continuously. In contrast, the friction coefficient curve at 800 °C exhibits only two stages, namely the initial running-in stage and a later steady stage. During the initial running-in stage, asperities on the contacting surfaces undergo plastic deformation and fracture under the applied load, resulting in an increased real contact area. During the mid-term steady stage (or the later steady stage at 800 °C), the formation of a stable tribo-oxidation layer at the friction interface establishes a dynamic equilibrium in the contact state. In the later destabilization stage, however, prolonged friction leads to heat accumulation, causing deterioration of the mechanical properties of the cladding layer material, subsequently leading to an abnormal rise in the friction coefficient. During friction at 800 °C, high-temperature oxidation leads to the formation of a tribo-oxidation layer covering the cladding layer surface. Under these conditions, the material is less affected by frictional heating, and therefore the friction coefficient curve does not exhibit a destabilization stage.

Figure 7e presents the average friction coefficient of the FG-Ni/WC/CeO_2_ cladding layers over the temperature range of 25–800 °C. The average friction coefficient exhibits significant variation with temperature. It is highest at 25 °C, and decreases with rising temperature, reaching its minimum value at 600 °C. However, a slight increase is observed at 800 °C. Figure 7f depicts the wear volume of the FG-Ni/WC/CeO_2_ cladding layers at different temperatures. The results reveal that each cladding layer exhibits its minimum wear volume at 25 °C and its maximum value at 800 °C. Compared to their respective minima at 25 °C, the wear volume at 800 °C increased by 604.32% for NWF5, 699.06% for NWF10, 960.79% for NWF15, and 1025.64% for NWF20. As the temperature increases from 600 °C to 800 °C, the wear volume increases sharply, and concomitantly, all cladding layers undergo a significant transition from mild wear to severe wear. Comparison with the average friction coefficients shown in Figure 7e indicates that the cladding layers exhibit relatively low average friction coefficients and wear volumes at 600 °C. Notably, NWF15 exhibits comparatively lower friction coefficients and wear volumes throughout the entire 25–800 °C temperature range.

**Figure 8:**
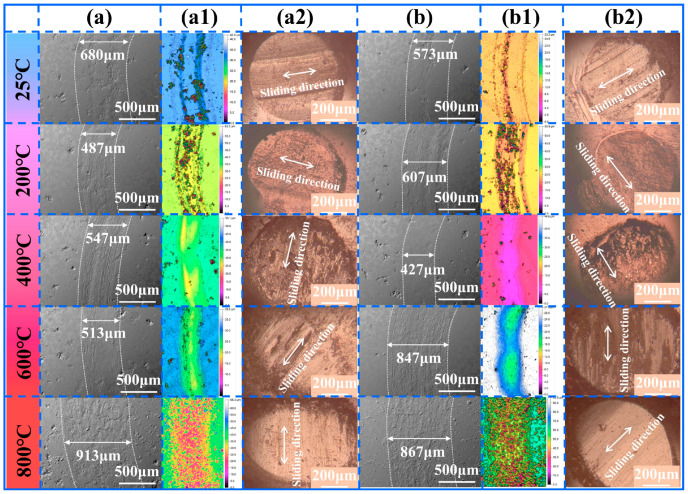
NWF5: (**a**) SEM morphology of wear track; (**a1**) corresponding three-dimensional morphology; (**a2**) corresponding OM morphology of Si_3_N_4_ counter ball. NWF10: (**b**) SEM morphology of wear track; (**b1**) corresponding three-dimensional morphology; (**b2**) corresponding OM morphology of Si_3_N_4_ counter ball.

**Figure 9:**
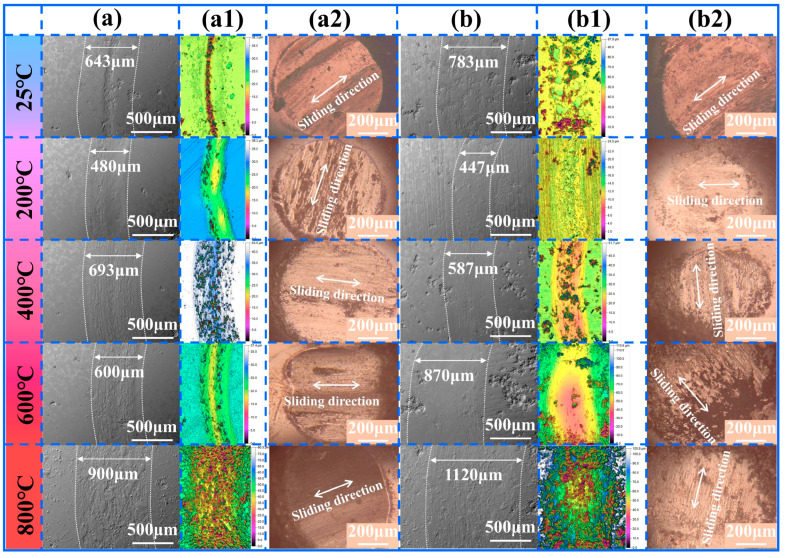
NWF15: (**a**) SEM morphology of wear track; (**a1**) corresponding three-dimensional morphology; (**a2**) corresponding OM morphology of Si_3_N_4_ counter ball. NWF20: (**b**) SEM morphology of wear track; (**b1**) corresponding three-dimensional morphology; (**b2**) corresponding OM morphology of Si_3_N_4_ counter ball.

Figure 8 and Figure 9 present the wear track SEM morphology, three-dimensional morphology of the wear track, and corresponding OM morphology of the Si_3_N_4_ counter balls’ wear scars for NWF5 and NWF10, and for NWF15 and NWF20, respectively. Analysis of the wear track SEM morphology reveals that the track width is significantly influenced by temperature, exhibiting an initial decrease followed by an increase with rising temperature. The minimum wear track width occurs at 200 °C for NWF5, NWF15, and NWF20, and at 400 °C for NWF10. At 800 °C, the track width reaches its maximum. Compared to their respective minimum widths at lower temperatures, the wear scar widths at 800 °C increased by 87.47% for NWF5, 103.04% for NWF10, 87.50% for NWF15, and 150.56% for NWF20. Analysis of the wear track’s three-dimensional morphology shows that at 25 °C and 200 °C, wear debris accumulation forms distinct ridges along the track edges. At 400 °C and 600 °C, the track surface appears relatively smooth. At 800 °C, the track surface exhibits pronounced undulations with evident signs of material spalling or adhesive transfer. OM analysis of the Si_3_N_4_ counter balls reveals that at 25 °C, linear protrusions aligned with the sliding direction are visible on the scar surface, resulting from the plowing effect induced by hard-phase particles (e.g., WC) within the FG-Ni/WC/CeO_2_ cladding layer on the Si_3_N_4_ counter balls. Within the 25–600 °C range, black-gray flaky transfer films cover the counter ball scar surface, indicating the occurrence of adhesive wear. At 800 °C, the counter ball surface appears relatively smooth and flat, but exhibits significant debris accumulation along the sliding direction.

### 3.4. Analysis of Wear Track and Debris

**Figure 10:**
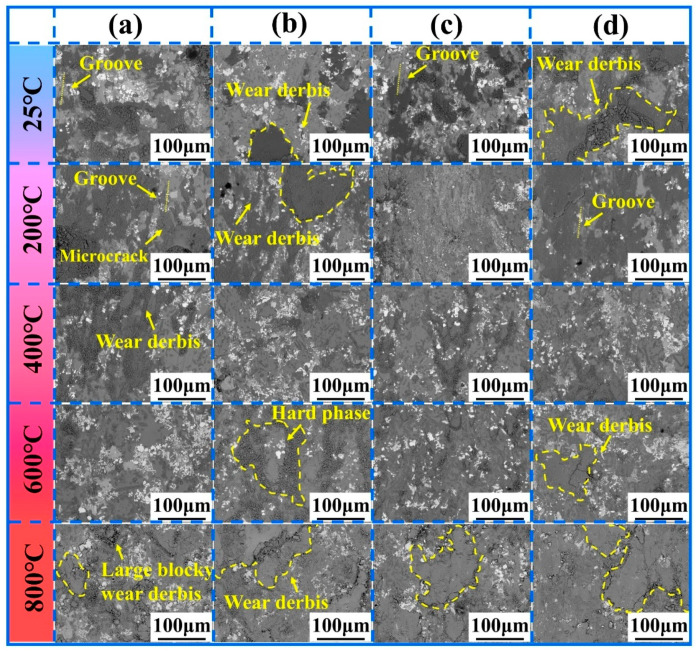
High-magnification morphologies of the wear tracks at different temperatures. (**a**) NWF5; (**b**) NWF10; (**c**) NWF15; (**d**) NWF20. The yellow dashed area demarcates the characteristic wear debris zone.

Figure 10a–d show the magnified SEM morphologies of the wear tracks for NWF5, NWF10, NWF15, and NWF20, respectively. At 25 °C, the cladding layer surface cracks and peels off under the continuous squeezing action of the Si_3_N_4_ counter ball, forming wear debris. The wear debris leaves distinct grooves on the cladding layer surface (Figure 10a,c at 25 °C), which is characteristic of typical abrasive wear [44]. The wear debris exhibits the typical feature of a dark gray matrix interspersed with white particles. Comparison with the microstructure of the cladding layers shown in Figure 5 confirms that the dark gray regions primarily consist of the matrix phase (e.g., γ-Ni), while the white particles primarily correspond to hard-phase particles (e.g., WC). This debris is compacted into and accumulates within surface depressions (e.g., NWF10 at 25 °C). Under cyclic stress, some accumulated debris is re-crushed and re-enters the friction process (e.g., NWF20 at 25 °C). At 200 °C, distinct microcracks (e.g., NWF5 at 200 °C) and grooves (e.g., NWF20 at 200 °C) are clearly observed on the surface of the accumulated debris. The presence of microcracks indicates that the debris is undergoing the crushing stage within the crushing–compaction cycle. The presence of grooves indicates that hard-phase particles are exposed at the friction interface and plow against the cladding layer surface. Compared to their condition at 25 °C, the surface morphology of accumulated debris transitions from relatively smooth to rough.

At a friction temperature of 400 °C, wear debris exhibited a discontinuous distribution with a relatively small coverage area on the wear track surface. This indicates that the oxidation of the cladding layer played a beneficial role in enhancing its wear resistance [45]. At 600 °C, wear debris distributed on the surface of the hard phase was compacted, while that dispersed on the nickel matrix surface surrounding the hard phase exhibited a loose distribution state (Figure 10b at 600 °C), suggesting that the addition of the hard phase effectively inhibits debris spalling. At 800 °C, the surfaces of all cladding layers exhibit wear debris that has been squeezed and smeared by the Si_3_N_4_ counter ball into contact surface depressions, and microcracks are prevalent on this debris. Larger wear debris fragments can be observed being detached from the interior of compacted debris (Figure 10a at 800 °C). Comparison with the friction coefficient curves in Figure 7a–d shows that no later destabilization stage occurs at 800 °C. Furthermore, the wear volume data in Figure 7f show a significant increase for all cladding layers at 800 °C. These observations collectively indicate that the persistent crushing–compaction cycle of debris throughout the friction process at 800 °C results in severe wear inflicted by the Si_3_N_4_ counter ball on the cladding layers.

Analysis of the friction coefficient curves and wear volume of Ni/WC/CeO_2_ cladding layers with different FG contents revealed that NWF15 exhibits relatively superior wear resistance. Therefore, the wear track microstructure of NWF15 was further analyzed.

**Figure 11:**
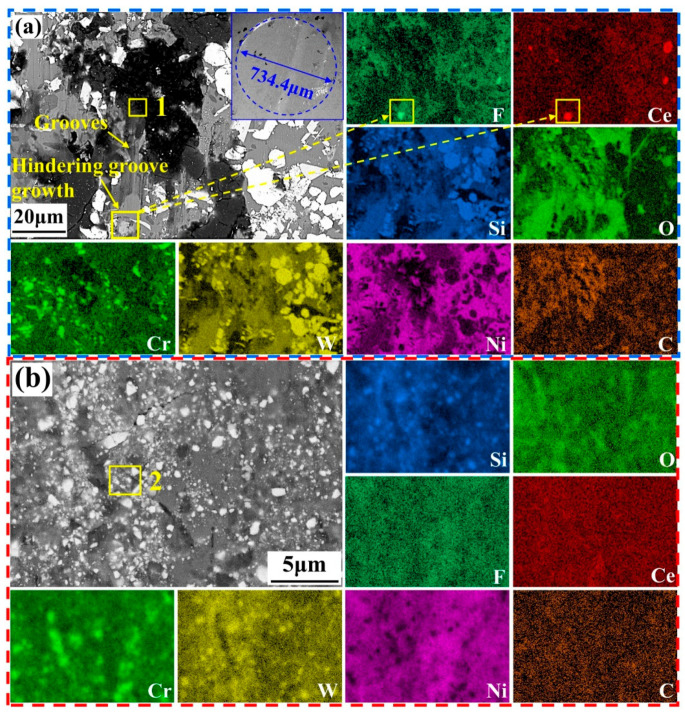
SEM image and EDS mapping of NWF15 wear track at (**a**) 25 °C and (**b**) 200 °C. Yellow boxes: numbered boxes correspond to EDS Micro-area analyses in Table 5; numberless box-arrow pairs denote EDS mapping zones with arrow indicating dominant elemental distribution.

Figure 11 shows the SEM microstructure and corresponding EDS mapping of the NWF15 wear track after testing at 25 °C and 200 °C. Figure 11a presents the microstructure of NWF15 after friction testing at 25 °C. Wear debris exhibiting a dark gray matrix interspersed with white particles is present on the cladding layer surface, along with the formation of plowing grooves. The elemental composition of the debris in Micro-area 1 is listed in Table 5. The Si content of the debris is 16.39 at%, significantly higher than the range of Si content (0.00–10.18 at%) within the NWF15 cladding layer (Table 3), indicating that the debris on the cladding layer surface contains material originating from the Si_3_N_4_ counter ball. During friction, debris peeled from the cladding layer surface enters the friction interface and is crushed into fine particles. This process exposes hard-phase particles (e.g., WC) at the friction interface. During relative sliding, these exposed hard-phase particles plow against the cladding layer surface (forming grooves) and simultaneously damage the Si_3_N_4_ counter ball. This explains the linear protrusions aligned with the sliding direction observed on the SEM image of the Si_3_N_4_ counter ball tested at 25 °C (within the blue boxed area in Figure 11a). Analysis of the XRD and TGA data from Figure 3, combined with EDS mapping in Figure 11a, confirms that the main phase at the location indicated within the yellow boxed region in Figure 11a is CeF_3_. Notably, CeF_3_ located at the terminus of the groove effectively halts its propagation, indicating that CeF_3_ contributes to reduced cladding layer wear.

To determine the primary chemical composition of the wear debris, the microscopic morphology of the wear debris generated from NWF15 at 200 °C was characterized. Figure 11b presents the wear track morphology of NWF15 at 200 °C. Compositional analysis of Micro-area 2 is presented in Table 5. The white particles are primarily composed of W (25.03 at%) and C (42.17 at%), respectively, consistent with the elemental constituents of WC, indicating that WC is the primary phase of these particles. Analysis of the EDS elemental distribution maps reveals that the primary elements in the dark gray regions are Ni and Cr. Combined with phase diagram knowledge and the cladding layer phase composition, it is inferred that the primary phases in these regions are Cr_7_C_3_, Cr_23_C_6_, and γ-Ni.

**Figure 12:**
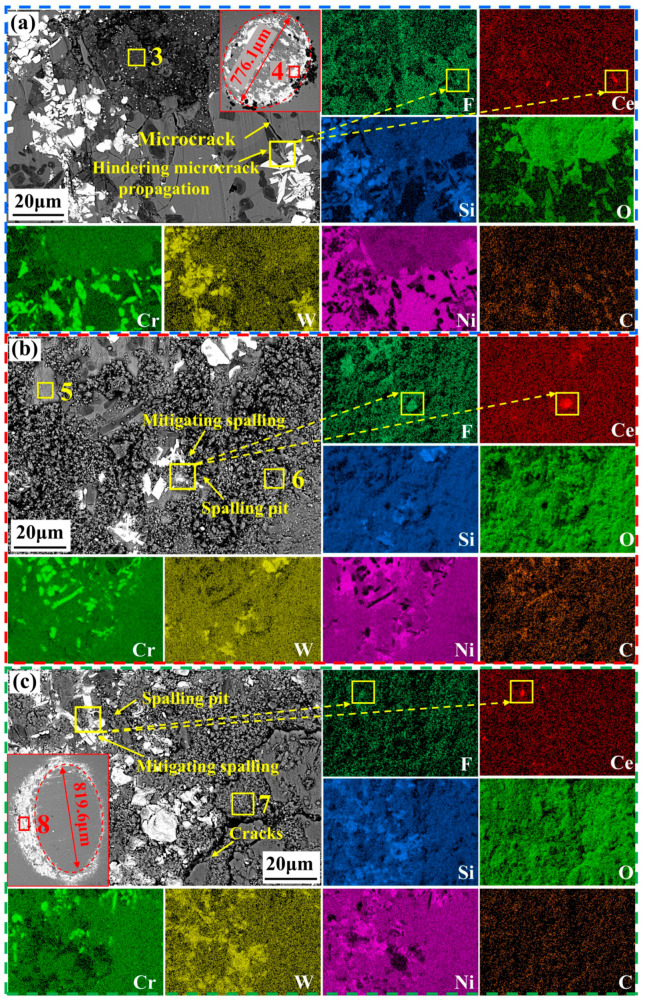
SEM image and EDS mapping of NWF15 wear track at (**a**) 400 °C, (**b**) 600 °C, and (**c**) 800 °C. Yellow boxes: numbered boxes correspond to EDS Micro-area analyses in Table 5; numberless box-arrow pairs denote EDS mapping zones with arrow indicating dominant elemental distribu-tion.

Figure 12 presents the wear track morphology and elemental distribution of NWF15 at 400 °C, 600 °C, and 800 °C. At 400 °C (Figure 12a), CeF_3_ at the yellow box location effectively protects the cladding layer against wear by hindering microcrack propagation. Within the wear debris (Micro-area 3 is listed in Table 5), the primary elements are O (38.05 at%) and Ni (22.32 at%), indicating the formation of a high-temperature oxide layer on the cladding layer surface. The morphology of the wear scars on the Si_3_N_4_ ball of the NWF15 specimen at 200 °C within the red box in Figure 12a is shown. The elemental composition of the debris in Micro-area 4, as listed in Table 5, is similar to that in Micro-area 3. Combined with the morphological features, this indicates that adhesive wear is the primary mechanism.

When the friction temperature reached 600 °C, the debris exhibited a spherical particle morphology (Figure 12b). Elemental analysis of Micro-area 5 (Table 5) revealed the highest concentrations of F (4.23 at.%) and Ni (58.33 at.%), indicating a γ-Ni matrix where the lubricating effect of CeF_3_ phases effectively mitigated further wear of the cladding. Within the yellow boxed region, CeF_3_ phases were distributed at the edges of spalling pits. As CeF_3_ phases were formed in situ during cladding and developed a 1.31 μm (Figure 5(b1)) diffusion layer with Ni, they were stably retained within the Ni matrix, thereby mitigating spalling and significantly enhancing the wear resistance of the cladding.

When the friction temperature increased to 800 °C, analysis of debris in Micro-area 7 revealed O content reached 60.18 at.%. Within the red box in Figure 12a, no debris covered the wear scars on the Si_3_N_4_ counter ball, while debris accumulated ahead of its sliding path. Elemental analysis of Micro-area 8 demonstrated W content increased from 13.04 at.% (Micro-area 4 at 400 °C) to 19.01 at.%, indicating severe surface oxidation of the cladding at 800 °C. The bulk spallation of the oxide layer from the friction surface during the friction process is identified as the primary cause of the dramatic increase in wear volume. Consequently, WC hard phases tended to detach from the matrix due to the shallow diffusion depth of W in the γ-Ni matrix (0.87 μm, Figure 5(b2)), generating debris and accelerating cladding wear. In contrast, CeF_3_ phases within the yellow box remained stable as at 600 °C, mitigating spalling to enhance wear resistance.

**Figure 13:**
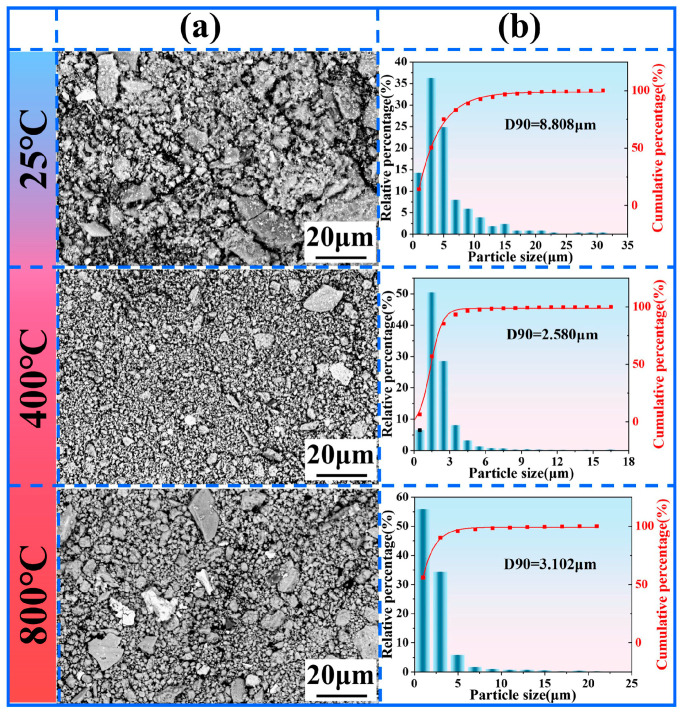
Wear debris of NWF15: (**a**) SEM image; (**b**) particle size distribution of corresponding wear debris.

Figure 13a shows the morphology of wear debris collected at the end of the friction test, while Figure 13b presents the corresponding particle size distribution statistics. Observation reveals that wear debris generated at 25 °C predominantly exhibits a flake-like morphology, with a minor presence of particle debris. The presence of flake-like debris indicates that the cladding layer undergoes plastic deformation under load, leading to the initiation of surface cracks. Subsequently, crack propagation results in the spallation of the cladding layer, forming wear debris. The average size of this flake-like debris is approximately 8.08 μm. At an elevated friction temperature of 400 °C, the wear debris primarily transitions to a loose particle morphology, with sporadic flake-like debris interspersed. The average debris size at this temperature significantly decreases to approximately 2.580 μm. These fine granular debris particles can be retained within the friction contact interface. By increasing the effective contact area, they contribute to a reduction in both the friction coefficient and wear rate [46,47,48]. When the friction temperature rises further to 800° C, the wear debris consists predominantly of relatively larger particles (D90 = 3.102 μm), accompanied by a significant amount of larger-sized irregular fragmentary debris. These large fragmentary debris particles are prone to being ejected from the contact interface during friction. This ejection promotes third-body abrasion, consequently leading to accelerated wear of the cladding layer [49,50,51].

**Figure 14:**
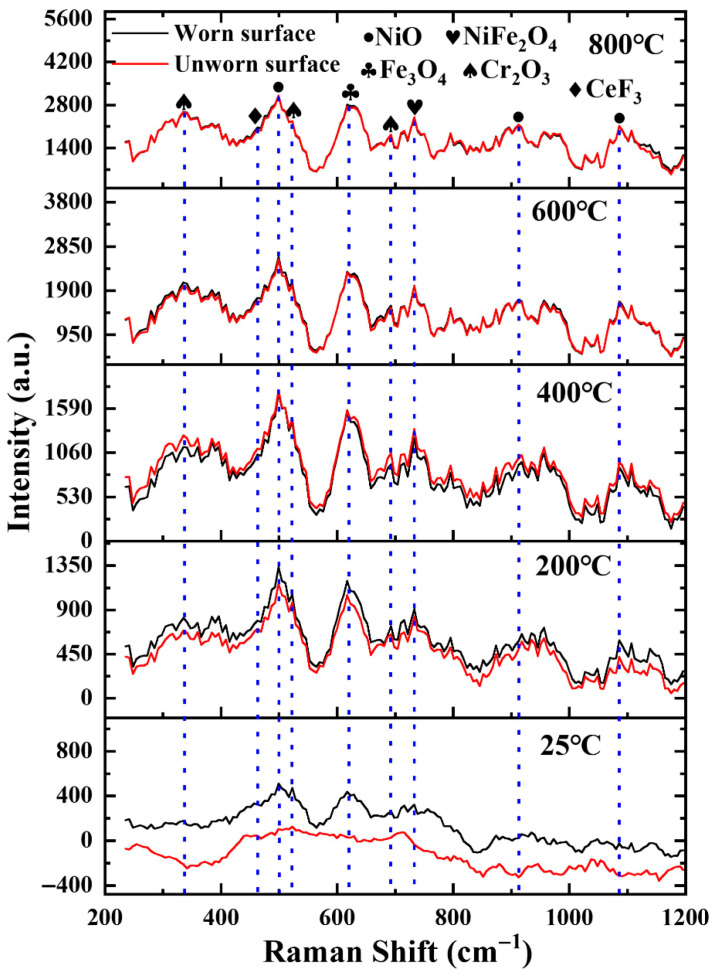
Raman analysis of NWF15. Blue dashed lines indicate characteristic Raman peak positions.

Figure 14 presents the Raman spectra of the worn region and the unworn region on the cladding layer surface. Raman analysis reveals that the oxides detected in the worn region during friction are NiO, NiFe_2_O_4_, Fe_3_O_4_,CeF_3_, and Cr_2_O_3_. At a friction temperature of 25 °C, characteristic Raman peaks of oxides are already detected in the worn region, whereas no discernible oxide peaks are observed in the unworn region. When the friction temperature increases to 200 °C, the intensity of the Raman characteristic peaks in the worn region is significantly higher than that in the unworn region, indicating that oxide formation in the worn region is predominantly driven by frictional heating at this stage. As the friction temperature rises further to 400 °C, the Raman peak intensity in the unworn region surpasses that in the worn region. This suggests a shift in dominance, where the contribution from ambient thermal effects to overall oxidation becomes predominant. Conversely, in the worn region, the intense thermo-mechanical coupling likely disrupts the integrity of the oxide layer or impedes its sufficient formation, resulting in diminished Raman signals. At elevated friction temperatures (600 °C and 800 °C), the Raman spectra from the worn and unworn regions become nearly identical. This observation indicates that under these high-temperature conditions, continuous and protective oxide layers form on the surfaces of both regions. Although friction is present, its effect on altering the oxide layer thickness is relatively minor. The dominant high-temperature oxidation process significantly overshadows the differentiating influence of mechanical friction on the surface microstructure and chemical state, leading to highly consistent oxidation characteristics across both regions.

### 3.5. Wear Mechanism

**Figure 15:**
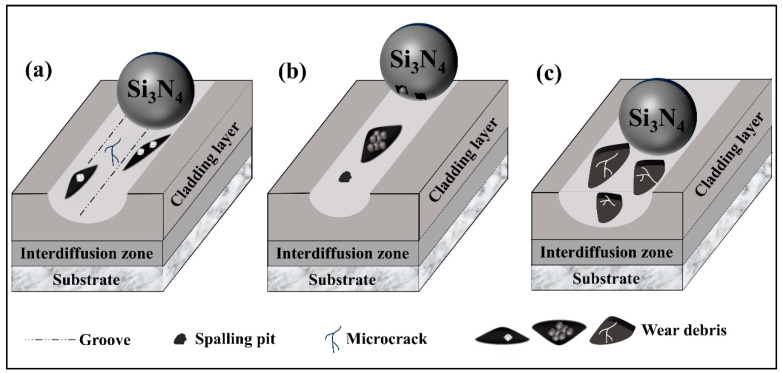
Schematic diagram of friction at different temperatures: (**a**) 25 °C; (**b**) 200–600 °C; (**c**) 800 °C.

Figure 15 illustrates the friction schematic of the cladding layer at different friction temperatures. At 25 °C, the extrusion of the Si_3_N_4_ counter ball on the cladding layer surface induces cracking and the formation of microcracks. The propagation of these microcracks results in the detachment of flake-like debris, which contains hard-phase WC particles. These detached WC particles not only scratch the cladding layer surface (forming grooves) but also score the wear scar surface of the Si_3_N_4_ counter ball. As friction progresses, the debris containing WC particles gradually embeds into the depressions on the cladding layer surface, thereby mitigating the severe wear caused by the WC particles. Consequently, at 25 °C, although the wear track width is relatively large, the wear volume remains comparatively low. The dominant wear mechanism at this stage is abrasive wear. When the friction temperature increases to 200–600 °C, wear debris is generated on both the cladding layer and Si_3_N_4_ counter ball wear track/scar surfaces. Under the influence of elevated temperature, this debris undergoes a gradual transformation into a granular form. These granular debris particles adhere to the friction contact interface, acting as friction-reducing media, which consequently reduces the wear volume of the cladding layer. The primary wear mechanism in this regime is adhesive wear.

At 800 °C, the oxide layer formed on the cladding layer surface fractures under the extrusion of the Si_3_N_4_ counter ball, generating initial debris that causes severe wear to the cladding layer surface. As friction proceeds, the debris composed of metallic oxides and cladding layer material undergoes a continuous cycle of fragmentation and re-agglomeration. During this process, the friction coefficient achieves a dynamically stable state. However, the presence and role of the metallic oxides lead to a significant increase in wear volume. The predominant wear mechanism under these conditions is oxidative wear.

### 3.6. Discussion

This study demonstrates that fluorinated graphite (FG) addition facilitates the in situ formation of a CeF_3_ self-lubricating phase, significantly enhancing the wide-temperature-range tribological performance of Ni/WC/CeO_2_ cladding layers. At 1.5 wt% FG (NWF15), a Ce:F atomic ratio of 1:7.06 closely approximates the stoichiometric 1:6 ratio, maximizing CeF_3_ phase formation efficiency. This promoted the formation of the finest average grain size (15.88 nm) and lowest porosity (0.141%) in NWF15, consequently yielding peak microhardness (1062.7 HV_0.2_) and optimal resistance to plastic deformation (H^3^/E^2^ = 0.0291). The cladding layers exhibited pronounced temperature-dependent wear behavior: abrasive wear dominated at 25 °C, adhesive wear prevailed from 200 to 600 °C, and oxidative wear became primary at 800 °C. Crucially, CeF_3_ effectively reduced wear damage by suppressing groove propagation and providing lubrication across 200–800 °C, with particularly pronounced effects at elevated temperatures. This mechanism provides a viable strategy for designing wide-temperature-adaptive wear-resistant cladding layers.

## 4. Conclusions

FG-reinforced Ni/WC/CeO_2_ cladding layers were successfully fabricated on 45 steel substrates using vacuum cladding technology, and their microstructure, phase composition, mechanical properties, friction, and wear behavior across a wide temperature range were systematically investigated. The main conclusions are summarized as follows:

(1) The addition of varying FG contents to the Ni/WC/CeO_2_ matrix during vacuum cladding led to the in situ formation of a CeF_3_ phase, which exhibited excellent high-temperature wear resistance and self-lubricating properties. The optimal formation efficiency of the CeF_3_ phase was achieved at an FG content of 1.5 wt% (Ce:F atomic ratio = 1:7.06). The corresponding NWF15 exhibited the smallest average grain size (15.88 nm) and the lowest porosity (0.141%).
(2) NWF15 exhibited the highest microhardness of 1062.7 HV 0.2. Compared to NWF5, this represents an increase of 10.64% in hardness. The H/E and H^3^/E^2^ ratios, indicators of resistance to elastic strain and plastic deformation, reached 0.0489 and 0.0291, respectively, corresponding to improvements of 20.74% and 78.53%. These results demonstrate the superior mechanical properties of NWF15.
(3) All cladding layers exhibited relatively low friction coefficients at 600 °C. However, a significant increase in wear severity was observed when the friction temperature rose from 600 °C to 800 °C. Throughout the entire tested temperature range (25–800 °C), the CeF_3_ phase effectively reduced the wear of the cladding layers primarily by inhibiting the propagation of grooves.
(4) Under friction at 25 °C, abrasive wear was the dominant mechanism. Within the 200–600 °C range, the primary wear mechanism shifted to adhesive wear. At 800 °C, oxidative wear prevailed. The oxides formed within the wear scars primarily consisted of NiO, NiFe_2_O_4_, Fe_3_O_4_, and Cr_2_O_3_.

## Figures and Tables

**Table 1:** Chemical composition of the Ni-based alloy powders (wt%) [38].

Element	C	B	Si	Cr	Fe	Ni
Mass fraction	0.7~1.1	3.0~4.0	3.5~5.0	15.0~17.0	≤5.0	Bal.

**Table 2:** Sample numbers and chemical composition.

Sample	Components (wt%)			
	Ni-Based Alloy	WC	CeO_2_	FG
NWF5	69.0	30	0.5	0.5
NWF10	68.5	30	0.5	1.0
NWF15	68.0	30	0.5	1.5
NWF20	67.5	30	0.5	2.0

**Table 3:** EDS point scanning results (at%) in Figure 5a,b.

Point	B	C	O	F	Si	Cr	Fe	Ni	Ce	W
1	28.28	21.35	0.59	4.26	0.05	6.60	2.51	25.45	0.01	10.90
2	24.00	23.35	0.00	4.83	2.20	7.31	1.94	28.49	0.12	7.76
3	24.27	34.99	1.38	2.60	0.99	21.86	1.40	11.95	0.02	0.54
4	4.81	54.83	2.30	0.00	0.00	0.67	0.22	3.42	0.08	33.61
5	34.35	15.63	0.23	3.48	0.00	6.28	2.35	24.02	0.03	13.63
6	23.35	18.70	0.00	5.27	10.18	0.85	0.45	41.07	0.01	0.14
7	33.22	14.70	0.00	4.27	0.02	2.39	2.07	43.08	0.00	0.25
8	0.00	12.51	1.55	63.85	0.23	0.34	0.10	2.23	19.15	0.03

**Table 4:** Mechanical properties of cladding layers. The ‘/’ denotes dimensionless ratios of identical units.

Sample	Nanohardness	Elasticity Modulus	H/E	H^3^/E^2^
	H (GPa)	E (GPa)	/	GPa
NWF5	9.94 ± 1.16	245.4 ± 4.21	0.0405	0.0163
NWF10	10.68 ± 1.23	252.2 ± 4.74	0.0423	0.0192
NWF15	12.17 ± 1.19	249.0 ± 4.38	0.0489	0.0291
NWF20	11.68 ± 1.33	258.1 ± 5.29	0.0453	0.0239

**Table 5:** EDS Micro-area scanning results (at%) of Figure 11 and Figure 12.

Micro-Area	C	O	F	Si	Cr	Fe	Ni	Ce	W
1	40.31	10.84	0.24	16.39	8.62	2.44	20.02	0.00	1.15
2	42.17	13.43	1.73	0.00	2.30	0.71	14.56	0.07	25.03
3	18.37	38.05	0.00	3.35	7.58	8.41	22.32	0.11	1.80
4	17.47	47.45	0.03	8.98	2.57	0.69	9.76	0.01	13.04
5	12.09	7.93	4.23	7.84	3.82	5.40	58.33	0.06	0.30
6	12.68	57.22	0.00	2.57	4.76	1.32	18.73	0.11	2.61
7	14.06	60.18	0.00	1.64	4.54	1.23	15.10	0.08	3.15
8	18.10	50.61	0.12	10.02	0.17	0.14	1.83	0.00	19.01

## Data Availability

The raw data supporting the conclusions of this article will be made available by the authors on request.
